# E-Service quality and customer satisfaction in online shopping: Implications for sustainable digital commerce

**DOI:** 10.1371/journal.pone.0362194

**Published:** 2026-11-06

**Authors:** Mohammed AL Awadh

**Affiliations:** 1 Department of Industrial Engineering, College of Engineering, King Khalid University, Abha, Saudi Arabia; 2 Center for Engineering and Technology Innovations, King Khalid University, Abha, Saudi Arabia; Universite Internationale de Rabat, MOROCCO

## Abstract

The rapid growth of e-commerce has intensified competition among online retailers, making e-service quality increasingly important for building customer trust, satisfaction, and sustainable customer–platform relationships. This study examines the relationships among e-service quality, customer trust, customer satisfaction, and customer loyalty using a second-order Structural Equation Modeling (SEM) framework. The five SERVQUAL dimensions such as empathy, responsiveness, reliability, assurance, and tangibles were modeled as first-order constructs reflecting the higher-order construct of service quality. Data were collected through an online questionnaire from 402 respondents with prior online-shopping experience. Confirmatory Factor Analysis (CFA) and SEM were employed to assess the measurement and structural models. The measurement model demonstrated satisfactory reliability, convergent validity, discriminant validity, and overall model fit. The structural results showed that service quality significantly and positively influenced customer trust and customer satisfaction, while customer trust significantly enhanced satisfaction. Trust also partially mediated the relationship between service quality and satisfaction, indicating that e-service quality improves satisfaction both directly and through increased customer confidence. The SERVQUAL dimensions contributed positively to overall service quality, with responsiveness and assurance showing particularly strong contributions, while empathy exhibited a comparatively moderate contribution. In contrast, customer loyalty had no significant direct effect on trust or satisfaction, and its indirect effect on satisfaction through trust was also nonsignificant. These findings demonstrate that reliable, responsive, secure, and well-designed digital services are important for strengthening trust and satisfaction in competitive e-commerce environments. By identifying trust as a key mechanism linking multidimensional e-service quality with customer satisfaction, the study contributes to SERVQUAL and digital-commerce literature and provides implications for the economic and relational sustainability of e-commerce platforms through more resilient and durable customer relationships.

## 1. Introduction

The rapid advancement of digital technologies and internet accessibility has transformed traditional retailing and accelerated the expansion of e-commerce. Online platforms enable organizations to conduct transactions efficiently and reach broader markets [1–4]. Consequently, online shopping has become a major component of modern retail markets, encouraging both emerging and traditional businesses to expand their operations into digital marketplaces [3,4]. However, their proliferation has also intensified competition, making e-service quality increasingly important for attracting and retaining customers [5,6]. In online shopping, customers evaluate service quality through website functionality, responsiveness, transaction security, communication effectiveness, and delivery reliability [7,8]. Deficiencies in these areas can lead to dissatisfaction, reduced trust, and customer switching behavior [9].

Alongside the expansion of digital commerce, sustainability has emerged as an important concern for e-commerce research and practice. Sustainable e-commerce encompasses environmental, economic, and social considerations associated with digital marketplaces, logistics, technological infrastructure, consumer behavior, and stakeholder relationships [10–12]. While much of this literature emphasizes environmental impacts and sustainable logistics, the long-term viability of digital commerce also depends on reliable service systems and durable customer relationships. Recent studies have therefore begun to connect service quality with sustainable digital services. Sustainable e-service quality has been associated with attributes such as security and efficiency [13], while SERVQUAL dimensions have been linked with the social sustainability of digital businesses [14]. Research in B2C e-commerce similarly connects service and logistics quality with satisfaction, trust, loyalty, eco-friendliness, and circular-economy objectives [15,16]. Digital service quality has also been recognized as an important component of sustainable digital transformation and customer-value creation [17].

Within this context, SERVQUAL provides an established framework for examining customer perceptions of service performance through five dimensions: empathy, responsiveness, reliability, assurance, and tangibles [18]. Although originally developed for conventional service environments, SERVQUAL has been widely adapted to e-commerce [19,20]. Responsiveness and reliability are particularly important for timely order processing, accurate information, dependable delivery, and problem resolution [21], while assurance-related attributes such as transaction security and platform credibility strengthen customer confidence [22]. Website design, interface quality, and product presentation further shape the overall digital-service experience [23].

Customer trust is particularly important because online transactions involve uncertainty, information asymmetry, privacy concerns, and perceived risk. Customers consequently rely on service-quality cues to evaluate platform credibility and reliability [24]. Trust can subsequently enhance satisfaction by reducing uncertainty and increasing confidence in digital transactions [25]. Its importance also extends to sustainability-oriented e-commerce research, where trust and satisfaction have been identified as important components of sustainable online-shopping relationships [26,27]. From this perspective, service quality does not directly represent environmental or social sustainability; rather, reliable, and customer-centered services can strengthen trust and satisfaction and thereby contribute to the economic and relational sustainability of digital commerce.

Despite extensive research on e-service quality, trust, satisfaction, and loyalty, important gaps remain. Previous studies frequently examine individual service-quality dimensions separately or operationalize service quality as a first-order construct, providing limited insight into how multiple SERVQUAL dimensions collectively represent overall e-service quality. Moreover, although sustainability-oriented studies increasingly recognize the importance of service quality, existing evidence remains fragmented across tourism, digital banking, logistics, e-government, and other digital-service settings [13,14,17]. Research in e-commerce has also connected logistics service quality with sustainability-related outcomes [15,16], but comparatively limited attention has been given to how the five SERVQUAL dimensions collectively form a higher-order e-service quality construct and influence customer satisfaction through trust within an integrated e-commerce framework. Furthermore, the role of customer loyalty as an antecedent of trust and satisfaction remains inconsistent and warrants further examination.

To address these gaps, this study develops a second-order Structural Equation Modeling (SEM) framework integrating empathy, responsiveness, reliability, assurance, and tangibles with customer trust, customer satisfaction, and customer loyalty in online shopping. The study examines the direct effects of service quality and loyalty on trust and satisfaction and evaluates the mediating role of trust. It contributes by (i) conceptualizing e-service quality as a higher-order SERVQUAL construct, (ii) clarifying the mediating role of trust and the antecedent role of loyalty, and (iii) extending sustainable e-commerce research by demonstrating how customer-centered service mechanisms can inform the economic and relational sustainability of digital platforms. Sustainability is therefore considered an implication of resilient and durable customer–platform relationships rather than a directly measured environmental or social construct.

## 2. Literature review

### 2.1. E-Service quality and SERVQUAL dimensions

Service quality is widely recognized as one of the most important determinants of customer perceptions and organizational performance. Unlike physical products, service quality is intangible and difficult to evaluate because customers assess services based on expectations and actual performance [28]. Within e-commerce environments, e-service quality reflects the extent to which online platforms effectively meet customer expectations throughout the digital transaction process [29]. Several service quality models have been developed to assess customer perceptions in different service contexts, including SERVQUAL, SERVPERF, E-S-QUAL, and HEDPERF [19,30]. Among these approaches, SERVQUAL remains an influential framework because of its multidimensional structure and applicability across different service settings [31].

SERVQUAL conceptualizes service quality through five dimensions: empathy, responsiveness, reliability, assurance, and tangibles [32,33]. Empathy refers to the ability of online platforms to provide personalized attention, customer support, and effective communication. Personalization strategies and customer-centered services improve customer engagement and perceived value. Responsiveness reflects the speed and effectiveness of customer service, order processing, issue resolution, and real-time updates. Reliability represents consistent service performance, including accurate product information, dependable delivery, and secure transactions. Assurance relates to professionalism, credibility, transaction security, and customer confidence in online platforms. Tangibles refer to website design, interface quality, product presentation, and packaging, which collectively influence user experience and purchasing decisions.

Previous studies consistently demonstrate that these dimensions collectively influence customer perceptions of e-service quality [33–35]. Consequently, recent research increasingly models service quality as a higher-order construct that integrates multiple dimensions within a unified framework [36,37]. This hierarchical perspective is particularly appropriate for online platforms because customers’ overall perceptions of service quality emerge from several simultaneous service encounters rather than from a single attribute.

More recent sustainability-oriented research extends this reasoning by demonstrating that service-quality characteristics can also contribute to sustainable digital-service systems. Khan et al. [13] developed a sustainable e-service quality framework in which security, efficiency, and other service attributes were used to evaluate digital platforms. Similarly, Umbreen et al. [14] examined the five SERVQUAL dimensions in relation to socially sustainable digital businesses, providing evidence that traditional service-quality capabilities can have broader organizational sustainability implications. Digital-quality research has also identified service quality as an important component of sustainable digital transformation and customer-value creation [17]. Collectively, these studies indicate that reliable and customer-centered service systems can contribute to the resilience and long-term viability of digital organizations.

Accordingly, the following hypotheses are proposed.

H1: Empathy loads positively on the higher-order Service Quality construct.

H2: Responsiveness loads positively on the higher-order Service Quality construct.

H3: Reliability loads positively on the higher-order Service Quality construct.

H4: Assurance loads positively on the higher-order Service Quality construct.

H5: Tangibles load positively on the higher-order Service Quality construct.

### 2.2. Service quality and customer trust

Trust is a critical determinant of online customer behavior because e-commerce transactions involve uncertainty, privacy concerns, and perceived risks. Customers are more likely to trust online platforms that demonstrate reliability, secure transactions, professional communication, and efficient service delivery [25,38]. High levels of service quality reduce uncertainty and strengthen customer confidence in online transactions. Accurate information, dependable delivery systems, responsive customer support, and secure payment mechanisms signal platform reliability and credibility, thereby enhancing customer trust.

Prior studies consistently report a positive relationship between e-service quality and customer trust in online shopping environments [39–42]. This relationship is also relevant to sustainability-oriented digital commerce. Sustainable digital relationships require customers to maintain confidence in the ability and willingness of platforms to provide reliable services over time. Recent sustainable e-commerce research identifies trust as an important component of customer responses to sustainable business and supply-chain practices [26,27]. Furthermore, evidence linking service quality with sustainable digital-business outcomes indicates that dependable and customer-centered services can contribute to stronger stakeholder relationships [14]. Therefore:

H6: Service Quality positively influences Customer Trust.

### 2.3. Service quality and customer satisfaction

Service quality is widely recognized as a direct antecedent of customer satisfaction in both traditional and digital service environments. In e-commerce, customers evaluate satisfaction based on factors such as website usability, delivery performance, customer support, and transaction security [43]. Previous empirical studies consistently demonstrate that high levels of e-service quality positively influence customer satisfaction [44–47]. Evidence from cross-border e-commerce demonstrates that logistics service quality is closely associated with customer satisfaction and that eco-friendliness can influence these relationships [16]. Research on B2C online retailing further integrates logistics service quality with customer satisfaction and broader circular-economy considerations [15]. These findings suggest that high-quality service systems can support not only immediate customer satisfaction but also more stable customer relationships. However, some studies suggest that this relationship may also operate indirectly through customer trust, highlighting the importance of examining mediation effects within integrated behavioral models [48–50]. Accordingly:

H7: Service Quality positively influences Customer Satisfaction.

### 2.4. Customer trust and customer satisfaction

Customer satisfaction represents customers’ overall evaluation of their shopping experience relative to their expectations. In online shopping environments, trust contributes significantly to customer satisfaction because trusted platforms reduce perceived risk and improve confidence during transactions. Previous studies indicate that customers who trust online platforms tend to report higher levels of satisfaction due to improved perceptions of reliability, security, and transaction efficiency [51–53]. Trust also enhances customer confidence regarding payment systems, delivery reliability, product authenticity, and the platform’s willingness to fulfill its commitments.

The relationship between trust and satisfaction has additional relevance within sustainable digital commerce. Jalil et al. [26] explicitly incorporated consumer trust and online-shopping satisfaction within a sustainability-oriented e-commerce framework, demonstrating the relevance of customer confidence to sustainable digital-business practices. Farhat et al. [27] similarly emphasized the role of trust in explaining favorable online-shopping evaluations within a sustainable e-commerce setting. These findings indicate that trust contributes not only to evaluations of individual transactions but also to the stable customer relationships necessary for the long-term viability of digital platforms. Thus:

H8: Customer Trust positively influences Customer Satisfaction.

### 2.5. Customer loyalty in e-commerce context

Customer loyalty reflects customers’ commitment to continue purchasing from a platform and maintaining long-term relationships with online retailers. Loyal customers may develop stronger trust and more positive evaluations of service performance due to repeated positive experiences. However, previous findings regarding the role of customer loyalty remain inconsistent. While some studies identify positive relationships between loyalty, trust, and satisfaction [54–56], others report weak or non-significant effects in highly competitive e-commerce environments where switching costs are relatively low [57–59].

These inconsistencies are particularly important from the perspective of sustainable digital commerce. Durable digital relationships require more than repeated transactions generated by temporary promotions, convenience, or price advantages. Customers can readily compare competing platforms, products, delivery options, and prices, meaning that behavioral repetition may not necessarily represent enduring relational commitment. Evidence from sustainable B2C retailing nevertheless indicates that service quality, satisfaction, trust, and loyalty can operate together within customer-centered sustainability frameworks [15]. This suggests that loyalty should be examined in conjunction with service quality and trust rather than assumed to independently guarantee stable customer relationships. Consequently, examining loyalty as a potential antecedent of trust and satisfaction provides an opportunity to determine whether existing customer commitment independently strengthens these outcomes when service-quality mechanisms are simultaneously considered. Therefore:

H9: Customer Loyalty positively influences Customer Trust.

H10: Customer Loyalty positively influences Customer Satisfaction.

### 2.6. Mediating role of customer trust

Customer trust represents a theoretically important mechanism through which service quality can influence subsequent customer evaluations. High-quality services reduce uncertainty by signaling competence, reliability, responsiveness, security, and professionalism. These perceptions strengthen customer confidence, which can subsequently contribute to more favorable evaluations of the overall shopping experience. Accordingly, service quality may influence customer satisfaction through both direct and indirect pathways. The direct pathway occurs when customers positively evaluate satisfactory service performance, whereas the indirect pathway operates when high-quality service strengthens trust, which subsequently enhances satisfaction. Previous research provides evidence for the mediating role of trust in relationships between e-service quality and customer outcomes [48–50].

This mechanism is also relevant to sustainable e-commerce. Jalil et al. [26] demonstrate the importance of trust within a framework incorporating sustainable supply-chain practices and online-shopping satisfaction, while Farhat et al. [27] further highlight trust as an important mechanism connecting customer perceptions with favorable online-shopping outcomes. In addition, research integrating service quality with socially sustainable digital-business outcomes suggests that relational mechanisms can mediate the translation of service capabilities into broader sustainable outcomes [14]. Collectively, these findings support the proposition that trust can function as a mechanism through which functional service performance contributes to stronger and more durable customer relationships.

Trust may similarly mediate the relationship between customer loyalty and customer satisfaction by transforming accumulated positive experiences into confidence and favorable evaluations of online platforms [23,60,61]. Nevertheless, because loyalty can be unstable in highly competitive online markets, the significance of this indirect mechanism requires empirical examination.

Examining these mediation pathways therefore contributes to understanding not merely whether service quality and loyalty affect customer satisfaction but also how these relationships operate. From a sustainable digital-commerce perspective, identifying trust as an intermediary mechanism can explain how customer-centered service capabilities translate into the confidence and satisfaction necessary for maintaining resilient platform–customer relationships. However, the present study does not treat sustainability as an additional latent construct; rather, the observed relationships are interpreted as providing implications for the economic and relational sustainability of digital commerce. Accordingly:

H11: Customer Trust mediates the relationship between Service Quality and Customer Satisfaction.

H12: Customer Trust mediates the relationship between Customer Loyalty and Customer Satisfaction.

Fig 1 presents the proposed conceptual framework, illustrating the five dimensions of service quality (H1–H5) and the hypothesized direct relationships among the study constructs (H6–H10). The framework also incorporates the indirect pathways through customer trust (H11–H12), highlighting its proposed mediating role in the relationships between service quality and customer satisfaction, and between customer loyalty and customer satisfaction.

**Fig 1:**
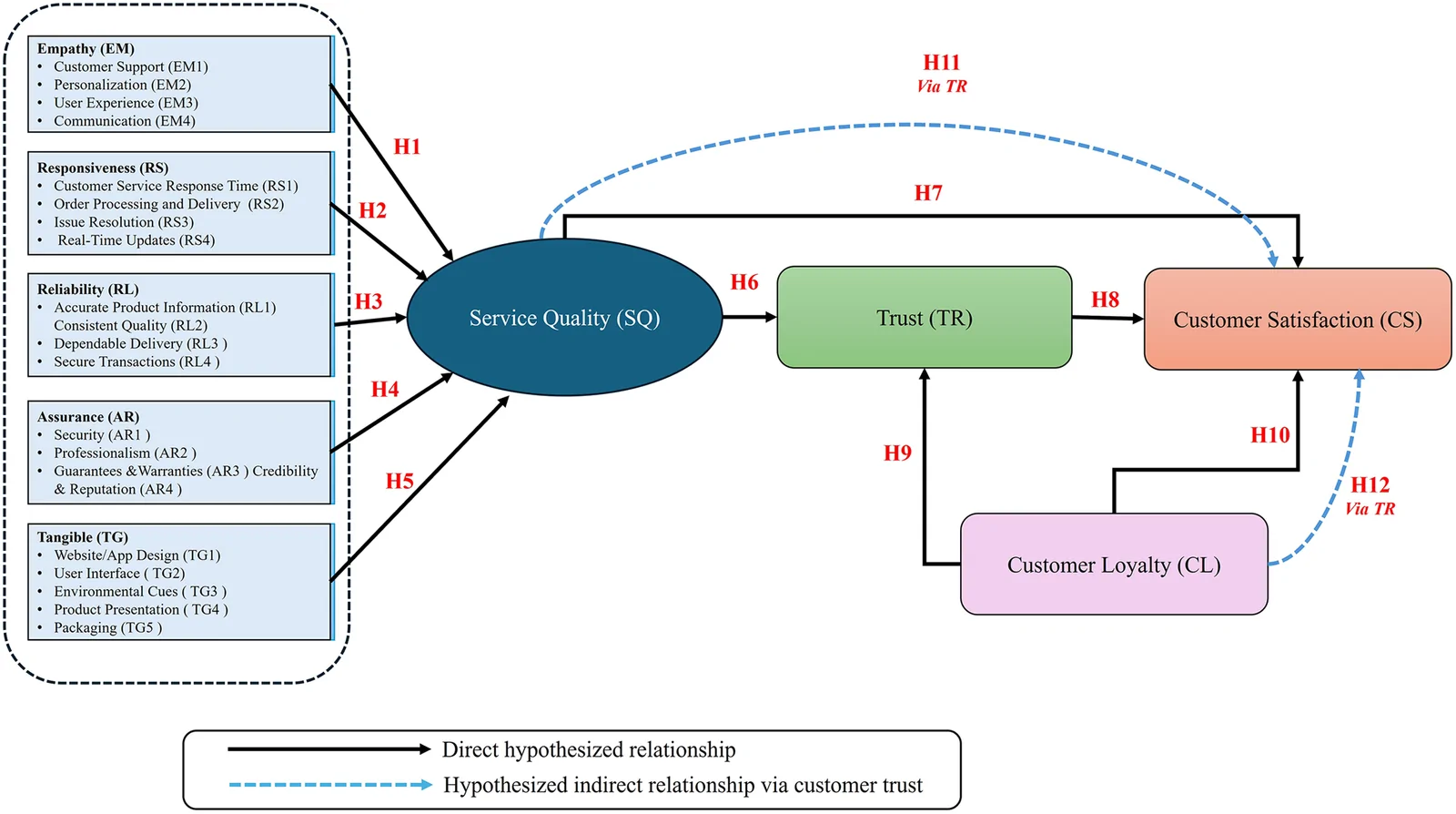
Initial Conceptual Framework of the Study. The framework illustrates the hypothesized relationships among service quality (SQ), customer trust (TR), customer satisfaction (CS), and customer loyalty (CL). Service quality comprises five SERVQUAL dimensions: empathy (EM), responsiveness (RS), reliability (RL), assurance (AR), and tangibles (TG). Solid arrows represent hypothesized direct relationships (H1–H10), whereas dashed arrows indicate hypothesized indirect relationships mediated by customer trust (H11–H12).

## 3. Materials and methods

This study adopted a quantitative research methodology to investigate the relationships among e-service quality, customer trust, customer satisfaction, and customer loyalty in online shopping environments. A second-order Structural Equation Modeling (SEM) framework was developed to evaluate both the measurement and structural relationships among the proposed constructs. The SERVQUAL dimensions, namely empathy, responsiveness, reliability, assurance, and tangibles, were modeled as first-order constructs contributing to the higher-order construct of service quality.

### 3.1. Research design

A cross-sectional survey design was employed to examine the hypothesized relationships among the latent constructs. A quantitative approach was considered appropriate because the study aimed to statistically evaluate relationships among multiple latent variables and test direct and indirect effects within an integrated theoretical framework.

The target population comprised individuals with prior experience purchasing products or services through online-shopping platforms. The conceptual framework integrated the five SERVQUAL dimensions with customer trust, customer satisfaction, and customer loyalty. Service quality was specified as a second-order construct reflected by empathy, responsiveness, reliability, assurance, and tangibles. The structural component of the model examined the direct effects of service quality and customer loyalty on customer trust and satisfaction, as well as the mediating role of customer trust.

### 3.2. Questionnaire development and respondents

A structured questionnaire (in English with no translation) was developed based on existing literature related to e-service quality, SERVQUAL, customer trust, customer satisfaction, and customer loyalty in e-commerce environments. Minor contextual modifications were introduced to ensure its suitability for online-shopping platforms. The questionnaire consisted of two main sections. The first section collected demographic information, including gender, age, educational level, frequency of online shopping, and preferred online-shopping platforms. The second section included measurement items for empathy (EM), responsiveness (RS), reliability (RL), assurance (AR), tangibles (TG), customer trust (TR), customer satisfaction (CS), and customer loyalty (CL). All items were measured using a five-point Likert scale ranging from 1 (Strongly Disagree) to 5 (Strongly Agree). The SERVQUAL dimensions were operationalized using multiple indicators adapted from previous studies. Empathy assessed personalized customer attention and communication effectiveness; responsiveness evaluated prompt service and issue resolution; reliability measured transaction consistency and delivery dependability; assurance reflected security and professionalism; and tangibles assessed website design and interface quality.

The target population comprised individuals with prior online-shopping experience. A non-probability convenience sampling approach was employed, and the questionnaire was distributed through social media, email, and other online communication channels. Data collection was conducted between 15 March and 20 July 2026. A total of 415 questionnaire responses were initially received. Before conducting the statistical analysis, the responses were screened for completeness and consistency. Responses containing incomplete information or inconsistent rating patterns were excluded. Consequently, 13 responses were removed during the screening process, resulting in a final sample of 402 valid responses for subsequent statistical analysis. Participation was voluntary, and respondents were informed about the purpose of the study, the confidentiality and anonymity of their responses, and their right to discontinue participation at any time. Ethical review and approval were waived, because the study involved an anonymous, voluntary, minimal-risk questionnaire survey that did not collect personally identifiable or sensitive information. Informed consent was obtained from all participants before completing the questionnaire through the consent information provided at the beginning of the survey.

### 3.3. Structural Equation Modeling (SEM)

The collected data were analyzed using Structural Equation Modeling (SEM) in Jamovi software (version 2.7.26), employing the SEMLj for confirmatory factor analysis, structural model estimation, and mediation analysis [62–64]. The SEM parameters were estimated using the Maximum Likelihood (ML) estimation method with the NLMINB optimization algorithm. SEM was selected because the proposed framework includes multiple dependent relationships, second-order constructs, and mediation effects involving service quality, customer trust, customer satisfaction, and customer loyalty. Initially, descriptive statistical analysis was performed to summarize respondent characteristics and examine the distributional properties of the collected data. Reliability and convergent validity of the measurement model were evaluated using Cronbach’s alpha (α), composite reliability (CR), and average variance extracted (AVE). Cronbach’s α and CR values greater than 0.70 were considered acceptable for internal consistency reliability, while AVE values exceeding 0.50 confirmed satisfactory convergent validity [65].

Confirmatory Factor Analysis (CFA) was subsequently conducted to evaluate the adequacy of the measurement model and examine the relationships between latent constructs and their observed indicators. Standardized factor loadings, standard errors, Z-values, and significance levels were assessed to confirm construct validity. In addition, discriminant validity was examined using the Heterotrait–Monotrait Ratio (HTMT), where HTMT values below 0.85 indicated adequate construct distinctiveness among the latent variables [66]. Following the validation of the measurement model, the structural model was evaluated to examine the hypothesized relationships among service quality, customer trust, customer satisfaction, and customer loyalty. Path coefficients (β), standardized estimates, and significance levels were used to test the proposed hypotheses. Furthermore, the mediating role of customer trust was examined through indirect effect analysis within the SEM framework.

Model adequacy was assessed using multiple goodness-of-fit indices, including the Comparative Fit Index (CFI), Tucker–Lewis Index (TLI), Incremental Fit Index (IFI), Normed Fit Index (NFI), Relative Fit Index (RFI), and Parsimony Normed Fit Index (PNFI). For the incremental fit indices, values approaching or exceeding 0.90 were considered indicative of acceptable model fit, with higher values representing better fit [65] PNFI was interpreted separately as a parsimony-adjusted fit measure, with comparatively higher values indicating a more favorable balance between model fit and complexity. The combined assessment of measurement quality, structural relationships, and overall model fit was used to determine the adequacy of the proposed second-order SEM framework.

## 4. Results

### 4.1. Respondent profile and data collection

A total of 402 valid questionnaire responses were collected from individuals with prior experience using online shopping platforms. The data were gathered through an online survey distributed using digital communication channels, including social media platforms and email. Before proceeding with the analysis, the collected responses were carefully screened to ensure completeness, consistency, and validity. Questionnaires containing substantial missing information or inconsistent response patterns were excluded, while all remaining responses were retained for further analysis. The obtained sample reflects active participation from online consumers with diverse demographic backgrounds and varying levels of online shopping experience. The relatively large number of responses indicates strong engagement with e-commerce platforms and provides a reliable representation of online consumer perceptions. The diversity of respondents also improves the comprehensiveness of the dataset and enhances the overall reliability of the study findings. Fig 2 illustrates the histogram of respondent distribution, showing the variation of respondents across different demographic categories. The distribution demonstrates balanced participation among different respondent groups, further supporting the representativeness and reliability of the collected dataset. The collected responses provided sufficient information to examine customer perceptions regarding service quality, trust, satisfaction, and loyalty within online shopping environments. The adequate sample size further strengthened the stability and consistency of the statistical analysis and supported the robustness of the subsequent analysis.

**Fig 2:**
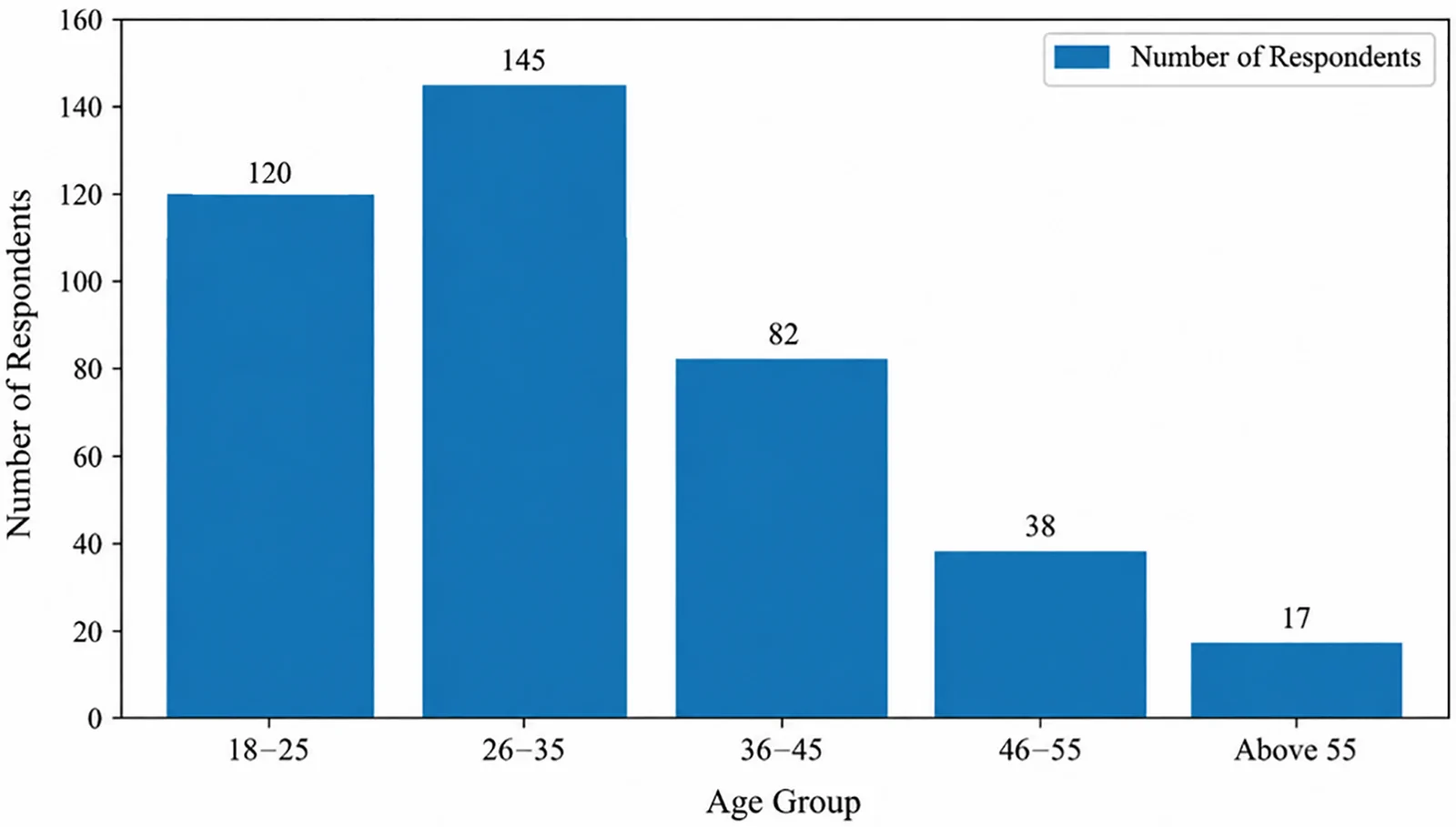
Respondent distribution for the collected questionnaire. The bar chart illustrates the age distribution of 402 respondents who participated in the online-shopping questionnaire. The horizontal axis represents age groups, while the vertical axis indicates the number of respondents. The largest proportion of respondents belongs to the 26–35-year age group (n = 145).

### 4.2. Confirmatory factor analysis

The confirmatory factor analysis results demonstrate a robust and reliable measurement model as summarized in Table 1. All observed indicators significantly load onto their respective latent constructs, namely EM, RS, RL, AR, and TG. The factor loadings are statistically significant (p < 0.001), with high Z-values ranging from 17.4 to 19.7, indicating strong associations between the observed variables and their underlying constructs. In addition, the relatively low standard errors (0.059–0.062) indicate stable and precise parameter estimation. The reliability analysis confirms strong internal consistency across all constructs. Cronbach’s α values range from 0.86 to 0.90, while CR values range from 0.85 to 0.89, both exceeding the recommended threshold of 0.70. These findings indicate that the measurement items consistently represent their corresponding latent variables.

**Table 1:** Summary of confirmatory factor analysis.

Factor	Indicator	Estimate	SE	Z	p	Cronbach’s α values	Composite reliability (CR)	Average variance extracted (AVE)
EM	EM1	1.1	0.0623	17.6	<.001	0.86	0.85	0.6
	EM2	1.11	0.062	17.9	<.001			
	EM3	1.09	0.0625	17.4	<.001			
	EM4	1.09	0.0625	17.4	<.001			
RS	RS1	1.09	0.0618	17.6	<.001	0.87	0.86	0.63
	RS2	1.15	0.0603	19.1	<.001			
	RS3	1.15	0.0603	19.1	<.001			
	RS4	1.11	0.0612	18.2	<.001			
RL	RL1	1.14	0.0603	18.9	<.001	0.89	0.87	0.67
	RL2	1.17	0.0595	19.7	<.001			
	RL3	1.15	0.0601	19.1	<.001			
	RL4	1.16	0.0598	19.3	<.001			
AR	AR1	1.1	0.0617	17.8	<.001	0.87	0.86	0.62
	AR2	1.1	0.0616	17.8	<.001			
	AR3	1.13	0.0607	18.6	<.001			
	AR4	1.14	0.0605	18.9	<.001			
TG	TG1	1.12	0.0607	18.4	<.001	0.9	0.89	0.63
	TG2	1.14	0.0601	19	<.001			
	TG3	1.11	0.061	18.1	<.001			
	TG4	1.13	0.0605	18.6	<.001			
	TG5	1.12	0.0607	18.4	<.001			

Convergent validity is also satisfactorily established. The AVE values range from 0.60 to 0.67, exceeding the recommended minimum value of 0.50. This indicates that each construct explains more than half of the variance of its indicators, confirming that the measurement items adequately capture the intended latent dimensions. Among the constructs, RL exhibits the strongest measurement properties, with the highest AVE (0.67) and CR (0.87), suggesting that accurate information, dependable delivery, and secure transactions are highly consistent dimensions within the online shopping context. TG also demonstrate strong reliability (α = 0.90), highlighting the importance of website design, interface quality, and product presentation in shaping customer perceptions of e-service quality.

CFA results provide strong empirical evidence supporting the reliability and convergent validity of the measurement model, confirming that the SERVQUAL dimensions are appropriately operationalized within the e-commerce environment.

### 4.3. Heterotrait–Monotrait Ratio (HTMT)

Discriminant validity was assessed using HTMT, which is considered a robust criterion for evaluating construct distinctiveness. The results are summarized in Table 2. The HTMT values range from 0.71 to 0.81, all of which are below the conservative threshold of 0.85, confirming adequate discriminant validity among the constructs. The highest HTMT value is observed between RS and AR (HTMT = 0.81), indicating a relatively strong conceptual association between these dimensions. This relationship is theoretically reasonable in online shopping environments, where prompt service and secure transaction processes jointly contribute to customer confidence and service evaluation. Nevertheless, the value remains within the acceptable threshold, confirming that the constructs remain empirically distinct. The remaining construct pairs exhibit moderate HTMT values ranging from 0.71 to 0.80, further supporting the conceptual uniqueness of empathy, responsiveness, reliability, assurance, and tangibles. These findings confirm that each SERVQUAL dimension captures a distinct aspect of e-service quality within the proposed model.

**Table 2:** Discriminant validity based on Heterotrait–Monotrait Ratio (HTMT).

	EM	RS	RL	AR	TG
EM	1.00	0.71	0.73	0.73	0.74
RS	0.71	1.00	0.80	0.81	0.77
RL	0.73	0.80	1.00	0.77	0.74
AR	0.73	0.81	0.77	1.00	0.80
TG	0.74	0.77	0.74	0.80	1.00

### 4.4. Model fit

The overall model fit was evaluated using multiple goodness-of-fit indices, all of which indicate excellent model adequacy as summarized in Table 3. The Comparative Fit Index (CFI = 0.993), Tucker–Lewis Index (TLI = 0.992), and Incremental Fit Index (IFI = 0.993) substantially exceed the recommended threshold of 0.90 and surpass the more stringent criterion of 0.95, demonstrating outstanding model performance. Similarly, the Normed Fit Index (NFI = 0.952) and Relative Fit Index (RFI = 0.945) further confirm the adequacy of the proposed model. The Parsimony Normed Fit Index (PNFI = 0.845) indicates that the model achieves strong explanatory capability while maintaining acceptable parsimony and avoiding unnecessary complexity. The consistency across multiple fit indices, including NNFI (0.992) and RNI (0.993), confirms the robustness and stability of the measurement and structural model. These findings indicate that the proposed SEM framework provides an excellent representation of the observed data and is appropriate for hypothesis testing and structural analysis.

**Table 3:** Model fit indices for SEM.

	Model
Comparative Fit Index (CFI)	0.993
Tucker-Lewis Index (TLI)	0.992
Bentler-Bonett Non-normed Fit Index (NNFI)	0.992
Relative Noncentrality Index (RNI)	0.993
Bentler-Bonett Normed Fit Index (NFI)	0.952
Bollen’s Relative Fit Index (RFI)	0.945
Bollen’s Incremental Fit Index (IFI)	0.993
Parsimony Normed Fit Index (PNFI)	0.845

### 4.5. Measurement model

The measurement model results as summarized in Table 4 further confirm the adequacy of the second-order SEM structure. At the first-order level, all observed indicators demonstrate strong and statistically significant relationships with their respective latent constructs. The standardized coefficients (β) range from 0.77 to 0.83 across the five SERVQUAL dimensions, with all paths significant at p < 0.001. These results indicate that the indicators consistently and reliably represent their corresponding constructs.

**Table 4:** Measurement model summary based on SEM.

Latent	Observed	Estimate	SE	95% Confidence Intervals		β	z	p
				Lower	Upper			
EM	EM1	1.00	0.00	1.00	1.00	0.78		
	EM2	1.01	0.06	0.89	1.14	0.79	16	<.001
	EM3	0.99	0.06	0.87	1.11	0.77	15.6	<.001
	EM4	0.99	0.06	0.87	1.11	0.77	15.6	<.001
RS	RS1	1.00	0.00	1.00	1.00	0.77		
	RS2	1.05	0.06	0.93	1.18	0.81	16.7	<.001
	RS3	1.06	0.06	0.94	1.18	0.81	16.9	<.001
	RS4	1.02	0.06	0.90	1.15	0.79	16.2	<.001
RL	RL1	1.00	0.00	1.00	1.00	0.80		
	RL2	1.03	0.06	0.92	1.14	0.83	18.4	<.001
	RL3	1.01	0.06	0.90	1.12	0.81	18	<.001
	RL4	1.01	0.06	0.90	1.12	0.82	18.1	<.001
AR	AR1	1.00	0.00	1.00	1.00	0.77		
	AR2	1.00	0.06	0.88	1.12	0.78	16.1	<.001
	AR3	1.03	0.06	0.91	1.15	0.80	16.7	<.001
	AR4	1.04	0.06	0.92	1.16	0.81	16.8	<.001
TG	TG1	1.00	0.00	1.00	1.00	0.79		
	TG2	1.02	0.06	0.91	1.14	0.81	17.5	<.001
	TG3	0.99	0.06	0.88	1.11	0.78	16.8	<.001
	TG4	1.01	0.06	0.90	1.13	0.80	17.2	<.001
	TG5	1.00	0.06	0.89	1.12	0.79	17	<.001
SQ	EM	1.00	0.00	1.00	1.00	0.82		
	RS	1.08	0.09	0.91	1.25	0.90	12.5	<.001
	RL	1.09	0.09	0.92	1.26	0.86	12.7	<.001
	AR	1.09	0.09	0.92	1.26	0.90	12.6	<.001
	TG	1.08	0.09	0.91	1.25	0.88	12.6	<.001

RL exhibits the strongest indicator loadings (β = 0.80–0.83), suggesting that customers place substantial emphasis on accurate product information, dependable delivery, and secure transactions when evaluating online shopping platforms. RS and AR also demonstrate strong loadings, reflecting the importance of efficient customer service, issue resolution, professionalism, and transaction security in e-commerce environments. At the second-order level, SQ is modeled as a higher-order construct reflected by EM, RS, RL, AR, and TG. The standardized coefficients range from 0.82 to 0.90, with all relationships statistically significant (p < 0.001). RS and AR exhibit the strongest contributions to SQ, followed by TG and RL, while EM demonstrates a comparatively moderate but still substantial contribution. These findings support the conceptualization of SQ as a multidimensional higher-order construct and confirm the theoretical suitability of the SERVQUAL framework within online shopping environments.

### 4.6. Structural and mediation effects results

The structural model results as tabulated in Table 5 reveal that SQ has a strong and statistically significant effect on customer trust (β = 0.449, p < 0.001), indicating that customers are more likely to trust online shopping platforms that provide reliable services, secure transactions, responsive communication, and professional support. This finding highlights the critical role of e-service quality in reducing uncertainty and enhancing confidence in digital commerce environments. TR also exerts a significant positive influence on CS (β = 0.285, p < 0.001), suggesting that trust enhances customers’ overall evaluations of their online shopping experiences. In addition, SQ directly influences CS (β = 0.133, p < 0.05), indicating that its effect operates through both direct and indirect mechanisms.

**Table 5:** Summary of structural and mediation effects.

Type	Path	Estimate	β	p-value	Hypothesis/Decision
Direct Effect	SQ → TR	0.704	0.449	< 0.001	H6/Supported
	CL → TR	0.076	0.076	0.092	H9/Not Supported
	TR → CS	0.285	0.285	< 0.001	H8/Supported
	CL → CS	0.056	0.056	0.226	H10/Not Supported
	SQ → CS	0.208	0.133	0.017	H7/Supported
Indirect Effect	SQ → TR → CS	0.201	0.128	< 0.001	H11/Supported
	CL → TR → CS	0.022	0.022	0.107	H12/Not Supported
Total Effect	SQ → CS	0.409	0.261	< 0.001	–
	CL → CS	0.078	0.078	0.106	–

The mediation analysis confirms that TR significantly mediates the relationship between SQ and CS (β = 0.128, p < 0.001). These findings suggest that high-quality online services enhance customer satisfaction not only directly but also indirectly through increased trust. Therefore, trust functions as an important psychological mechanism linking service quality perceptions to customer satisfaction outcomes. In contrast, CL does not significantly influence TR (β = 0.076, p > 0.05) or CS (β = 0.056, p > 0.05). Similarly, the indirect effect of CL through TR is not statistically significant (β = 0.022, p > 0.05). These findings indicate that customer loyalty does not operate as a primary antecedent within the proposed model. The non-significant loyalty relationships may reflect the highly competitive nature of online shopping environments, where customers frequently switch between platforms based on price, convenience, promotions, or delivery efficiency rather than long-term attachment. In summary, the results indicate that SQ and TR constitute the primary mechanisms influencing CS in online shopping platforms, while CL demonstrates a comparatively limited role within the proposed framework. The final framework is presented in Fig 3.

**Fig 3:**
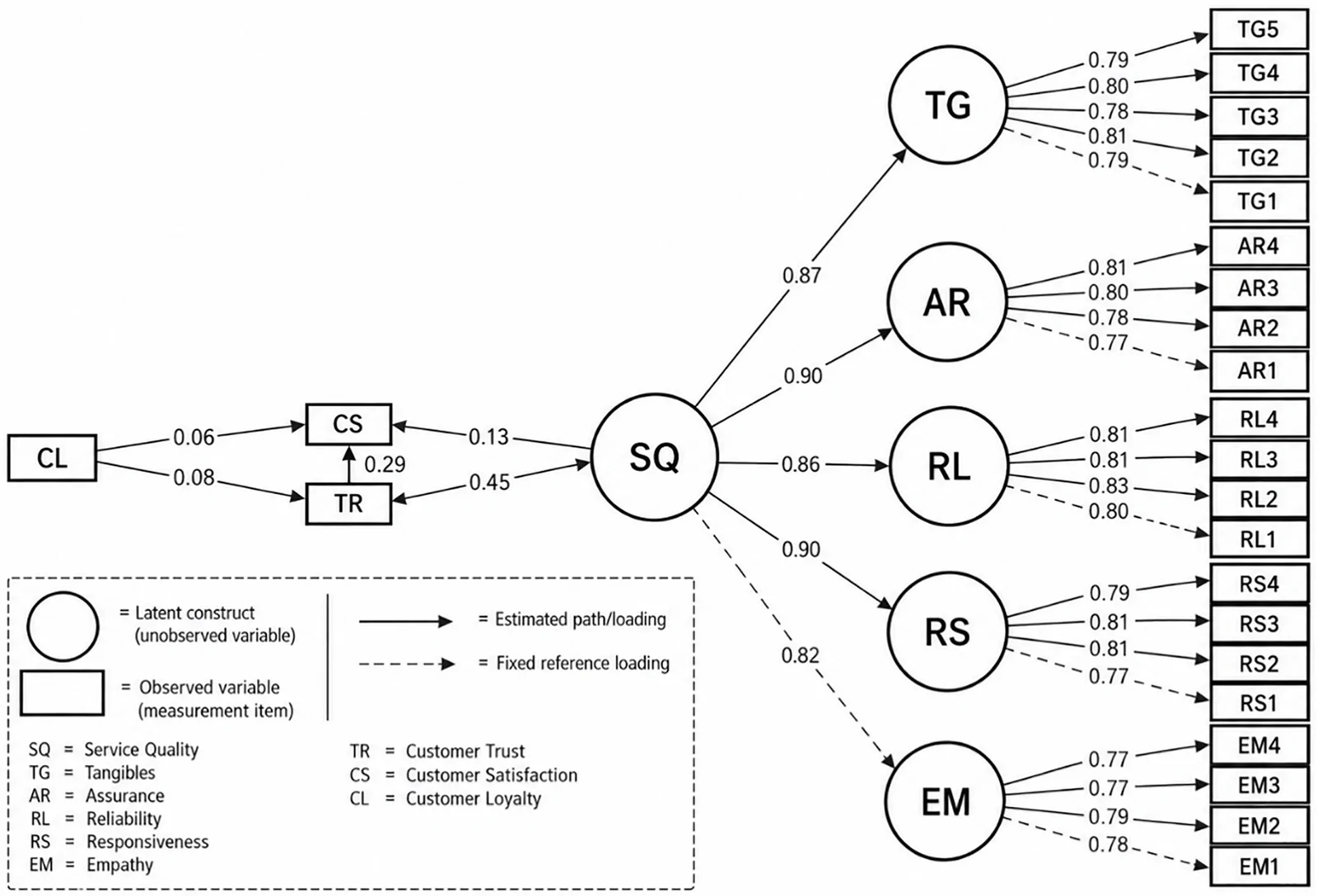
Structural equation model (SEM) path diagram showing standardized path coefficients (β) values. Circles represent latent constructs, while rectangles indicate observed variables, including measurement indicators and single-item variables. Solid arrows represent estimated paths or factor loadings, whereas dashed arrows indicate fixed reference loadings used for model identification. Numerical values indicate standardized coefficients (β). SQ = service quality; EM = empathy; RS = responsiveness; RL = reliability; AR = assurance; TG = tangibles; TR = customer trust; CS = customer satisfaction; CL = customer loyalty.

## 5. Discussion

The findings provide further evidence for the applicability of the SERVQUAL framework in digital commerce and demonstrate that e-service quality is a multidimensional construct shaped by customers’ evaluations of different aspects of the online service experience. The strong measurement properties of the model indicate that empathy, responsiveness, reliability, assurance, and tangibles collectively represent customers’ perceptions of e-service quality. Among these dimensions, reliability emerged as an important component, suggesting that customers place considerable value on dependable service delivery, accurate information, transaction consistency, and secure online interactions. This finding is consistent with previous studies identifying reliability as an influential determinant of customers’ evaluations of online services because dependable performance reduces uncertainty and perceived transaction risk [53,67]. It is also supported by recent sustainability-oriented service research showing that reliability and efficiency are important components of sustainable digital-service systems [13]. Similarly, research on sustainable e-banking identifies reliability and efficiency as particularly important determinants of customer satisfaction, further demonstrating the importance of dependable digital services for maintaining effective customer relationships.

Tangibles also made a strong contribution to the higher-order service-quality construct, highlighting the importance of website design, interface quality, and product presentation in shaping customers’ evaluations of online platforms. Because customers cannot physically interact with products, employees, or conventional service facilities during many stages of online purchasing, digital interfaces become important observable cues through which service competence and professionalism are evaluated. This interpretation is consistent with previous research demonstrating that visually appealing, functional, and user-friendly websites improve perceived service quality and customer engagement [68–70]. From a broader digital-sustainability perspective, effective digital interfaces may also support more efficient customer–platform interactions by improving accessibility, information exchange, and the overall quality of the digital experience [71].

Responsiveness and assurance also contributed strongly to service quality, indicating that customers value prompt support, effective problem resolution, professional service, and secure transactions. These findings are consistent with previous research showing that responsiveness and assurance are particularly important in online environments, where the absence of face-to-face interaction increases customers’ dependence on timely communication and credible security mechanisms [71,72]. Recent research on sustainable e-service quality similarly identifies security and efficiency among the important attributes influencing evaluations of digital services [13]. Thus, the present findings reinforce the view that the long-term quality of digital commerce depends not only on technological functionality but also on customers’ confidence that platforms will respond effectively and protect their transactions.

In comparison, empathy made a relatively moderate contribution to overall service quality. This may reflect the predominantly technology-mediated nature of online shopping, where customers often prioritize convenience, reliability, speed, and functionality over interpersonal interaction. Similar observations were reported by Thorat and Parlikar [73], who argued that customers in digital marketplaces may attach greater importance to operational efficiency and convenience than to personalized interaction. Nevertheless, empathy should not be considered unimportant. Recent research connecting the five SERVQUAL dimensions with socially sustainable digital businesses indicates that human-centered service attributes can remain relevant to broader stakeholder relationships [14]. Online retailers may therefore need to balance efficient digital service delivery with appropriate personalization and customer support rather than treating the two as competing objectives.

The structural findings further demonstrate the central role of service quality in developing customer trust. Customers are more likely to trust platforms that provide dependable services, secure transactions, effective communication, and responsive customer support. This result supports previous research showing that e-service quality reduces perceived uncertainty and strengthens confidence in online transactions [63,74,75]. In digital commerce, where customers frequently encounter information asymmetry, privacy concerns, and transaction risk, service-quality cues provide evidence of platform competence and credibility. The significant service quality–trust relationship is therefore theoretically important because it demonstrates that trust is not generated independently of customers’ service experiences but is partly developed through repeated evaluations of platform performance.

This relationship also provides an important connection with the emerging sustainable e-commerce literature. Jalil et al. [26] identified consumer trust as an important component of sustainable e-commerce relationships involving green supply-chain management and online-shopping satisfaction, while Farhat et al. [27] similarly emphasized trust within a sustainability-oriented online-shopping framework. Furthermore, studies of socially sustainable digital businesses have linked service-quality capabilities with broader sustainable organizational relationships [14]. The present findings complement this literature by showing that, within online shopping, a multidimensional higher-order service-quality construct can serve as an important antecedent of customer trust. In this sense, reliable and trustworthy service relationships may contribute to the relational resilience and long-term viability of digital commerce, although the present study does not directly measure environmental or social sustainability outcomes.

The significant positive relationship between customer trust and customer satisfaction further confirms the importance of trust in shaping customers’ evaluations of online-shopping experiences. Trust can reduce concerns regarding payment security, delivery reliability, product authenticity, and the ability of a platform to fulfill its commitments, thereby enabling customers to evaluate their overall experience more favorably. This finding is consistent with previous research identifying trust as an important psychological mechanism underlying positive customer evaluations in e-commerce [76,77]. It is also consistent with sustainability-oriented e-commerce research linking consumer trust with online-shopping satisfaction [26,27]. Accordingly, trust appears to perform both a transactional function—by reducing uncertainty during individual purchases—and a relational function by strengthening customers’ confidence in continued interactions with an online platform.

Service quality also exerted a significant direct effect on customer satisfaction. This indicates that customers’ satisfaction is influenced not only through trust formation but also directly through their evaluations of service performance, including website functionality, transaction efficiency, customer support, and fulfillment. This result is consistent with previous findings that higher e-service quality enhances customer satisfaction in online-shopping environments [57,73]. It also corresponds with research on cross-border e-commerce showing meaningful relationships between logistics service quality and customer satisfaction, including the role of eco-friendly logistics practices [70]. More broadly, studies of sustainable digital services have shown that reliable, efficient, and responsive service provision is associated with favorable customer evaluations. These converging findings indicate that maintaining service quality remains fundamental to both immediate customer satisfaction and the development of more durable digital relationships.

The mediation analysis provides additional evidence regarding the role of trust within the proposed framework. The significant indirect effect of service quality on customer satisfaction through trust indicates that high-quality online services improve customer satisfaction both directly and indirectly by enhancing customer confidence in the platform. This finding supports theoretical arguments suggesting that trust functions as an important intermediary mechanism linking service quality perceptions to favorable customer outcomes in online environments [78]. Similar mediation effects were reported in prior SEM-based e-commerce studies, which found that trust partially mediates the relationship between service quality and customer behavioral outcomes [79,80].

An important contrasting result concerns customer loyalty. Loyalty did not significantly predict either customer trust or customer satisfaction, and its indirect effect on satisfaction through trust was also nonsignificant. These findings differ from studies reporting positive associations among loyalty, trust, and satisfaction [81,82], but are consistent with evidence suggesting that loyalty may not necessarily operate as a strong antecedent in highly competitive digital markets characterized by low switching costs [83,84]. Online consumers can readily compare platforms and change providers according to price, promotions, delivery speed, convenience, product availability, and other situational considerations. Consequently, previous purchasing behavior or attachment to a platform may not independently generate greater trust or satisfaction when customers’ current service experiences are considered.

This finding requires careful interpretation because it does not imply that customer loyalty is irrelevant to e-commerce sustainability. Rather, it suggests that the direction of influence may be more complex than assuming that existing loyalty automatically produces trust and satisfaction. Sustainable B2C retail research has incorporated service quality, satisfaction, trust, and loyalty within interconnected customer-response mechanisms [15]. The present results suggest that online retailers seeking durable customer relationships should not rely solely on established customer loyalty; instead, they may need to continually reinforce loyalty through reliable service delivery, trust-building, and satisfactory customer experiences. This interpretation is particularly relevant in digital marketplaces where customers face relatively few barriers to switching between competing platforms.

Taken together, the findings identify service quality and trust as the principal mechanisms associated with customer satisfaction in the proposed model. Reliable service delivery, responsive communication, transaction assurance, and effective digital interfaces strengthen the overall quality of customers’ online-shopping experiences, while trust provides an important pathway through which these service evaluations translate into satisfaction. These results also complement emerging research connecting digital service quality with sustainable digital transformation [17], sustainable e-service systems [13], socially sustainable digital businesses [14], and sustainability-oriented B2C commerce [15,16,26,27]. Accordingly, the sustainability implication of the present study lies primarily in the economic and relational durability of digital commerce: platforms that consistently provide high-quality and trustworthy services are better positioned to sustain satisfactory customer relationships over time. However, because environmental and social sustainability outcomes were not directly measured, these broader sustainability implications should be interpreted as theoretically informed extensions of the empirical findings rather than as directly tested causal effects.

## 6. Conclusions

This study developed a second-order SEM framework to examine the relationships among e-service quality, customer trust, customer satisfaction, and customer loyalty in online-shopping environments. The findings confirm that empathy, responsiveness, reliability, assurance, and tangibles collectively represent e-service quality, with responsiveness, assurance, reliability, and tangibles showing particularly strong contributions. Service quality significantly influenced both customer trust and satisfaction, while trust also significantly enhanced satisfaction and partially mediated the relationship between service quality and satisfaction. In contrast, customer loyalty did not significantly influence trust or satisfaction, and its indirect effect through trust was also nonsignificant.

These findings highlight the importance of reliable service delivery, secure transactions, responsive communication, and effective digital interfaces for strengthening customer confidence and satisfaction. From a sustainable digital-commerce perspective, such service capabilities can support the economic and relational sustainability of online platforms by fostering more resilient and durable customer–platform relationships. However, the study does not directly measure environmental or social sustainability; therefore, these implications should be interpreted as extensions of the observed service-quality and relationship mechanisms rather than as direct sustainability effects.

The study has several limitations. The cross-sectional research design restricts causal and longitudinal interpretation, while the use of non-probability convenience sampling may limit the generalizability of the findings. Therefore, the results should primarily be interpreted in relation to the surveyed respondents rather than being generalized to the entire online-shopping population. In addition, the model focuses on service quality, trust, satisfaction, and loyalty and does not incorporate other potentially relevant factors such as perceived value, website usability, price sensitivity, switching costs, or cultural differences. Future research should employ longitudinal and cross-country designs, use more diverse sampling approaches, and extend the model by incorporating explicit sustainability-related constructs such as green logistics, sustainable packaging, eco-friendliness, sustainable consumption, and social responsibility to directly examine the environmental, social, and economic dimensions of sustainable e-commerce.
